# Examining the Evidence for Chytridiomycosis in Threatened Amphibian Species

**DOI:** 10.1371/journal.pone.0023150

**Published:** 2011-08-03

**Authors:** Matthew Heard, Katherine F. Smith, Kelsey Ripp

**Affiliations:** Department of Ecology and Evolutionary Biology, Brown University, Providence, Rhode Island, United States of America; Australian Wildlife Conservancy, Australia

## Abstract

Extinction risks are increasing for amphibians due to rising threats and minimal conservation efforts. Nearly one quarter of all threatened/extinct amphibians in the IUCN Red List is purportedly at risk from the disease chytridiomycosis. However, a closer look at the data reveals that *Batrachochytrium dendrobatidis* (the causal agent) has been identified and confirmed to cause clinical disease in only 14% of these species. Primary literature surveys confirm these findings; ruling out major discrepancies between Red List assessments and real-time science. Despite widespread interest in chytridiomycosis, little progress has been made between assessment years to acquire evidence for the role of chytridiomycosis in species-specific amphibian declines. Instead, assessment teams invoke the precautionary principle when listing chytridiomycosis as a threat. Precaution is valuable when dealing with the world's most threatened taxa, however scientific research is needed to distinguish between real and predicted threats in order to better prioritize conservation efforts. Fast paced, cost effective, in situ research to confirm or rule out chytridiomycosis in species currently hypothesized to be threatened by the disease would be a step in the right direction. Ultimately, determining the manner in which amphibian conservation resources are utilized is a conversation for the greater conservation community that we hope to stimulate here.

## Introduction

Recent research has suggested that extinction risks are increasing for vertebrates due to high levels of threat coupled with unsuccessful conservation efforts to mitigate species loss [1]–[4]. Of particular concern are amphibians, approximately 41% of which are classified as ‘threatened’ (e.g. vulnerable, endangered or critically endangered) by the International Union for Conservation of Nature Red List (IUCN Red List) [4], [5]. A 2010 synthesis of the Red List concluded that amphibians are more threatened than either birds or mammals with index values (aggregated measures of extinction risk) declining more than three percent from 1980 to 2004; a deterioration equivalent to 662 species each moving one Red List category closer to extinction during this time [4].

The decline of amphibians is among the world's most compelling conservation issues [3], [6], [7] and the disease chytridiomycosis, caused by the fungal pathogen *Batrachochytrium dendrobatidis* (*Bd*), is widely believed to play a role in these declines due to its rapid spread, global distribution, broad diversity of host species, and high virulence [3], [6], [8]–[11]. In Latin America alone, *Bd* has been implicated in the possible extinctions of ∼27% of the region's 113 species of *Atelopus* harlequin toads [11], [12]. To conserve amphibians from chytridiomycosis and other threats, scientists have called for action plans similar to those that have been effective for birds and mammals [4]. Though nearly all threatened species are at risk from multiple causal factors, the most successful conservation actions have explicitly targeted individual threats: invasive species eradications from islands to save birds and mammals from non-native predators [13], [14], site-specific hunting bans to preserve birds in Brazil, and international legislation to protect marine mammals, like the Vicuña, from bycatch [4], [15], [16]. Designing focused action plans like these is a complex and case-specific process that relies heavily on scientific evidence to validate threats and quantify the magnitude of impact. The IUCN Red List represents the worldwide standard for evaluating extinction risks [17], [18] and has been used repeatedly to assess the impact of individual and multiple threats in the loss of global diversity and for specific taxonomic groups [19]–[21].

**Figure 1:**
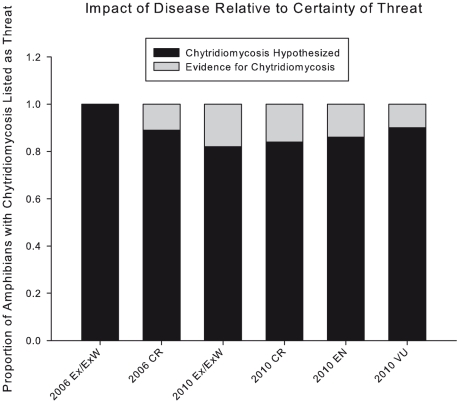
Proportion of amphibian species with disease reported as a threat by the Red List, distinguished by those with supporting evidence and those where disease is only hypothesized. Extinct (Ex) and Extinct in the Wild (ExW) pooled, Critically Endangered (CR), Endangered (EN) and Vulnerable (VU). 2006 data is adapted from Smith et al. [21] and includes amphibians threatened generally by disease (not distinguishing those threatened by chytridiomycosis/*Bd*). 2010 data includes amphibians specifically threatened by chytridiomycosis/*Bd* (97% of disease-threatened amphibian species). Only two species account for the 2006–2010 increase in the proportion of Ex/ExW amphibians with evidence of disease/chytridiomycosis: *Anaxyrus baxteri* and *Nectophrynoides asperginis* are both extinct in the wild, each having natural populations with confirmed presence of *Bd* since 2006). Evidence for Chytridiomycosis: chytridiomycosis is a confirmed threat and/or the fungus *Batrachochytrium dendrobatidis* (*Bd*) has been reported in at least one of the species' populations and not confirmed as non-pathogenic. Chytridiomycosis Hypothesized: no evidence exists to support chytrid as a threat, *Bd* has not been detected in the species, or the species is determined asymptomatic when infected.

In 2006, Smith *et al.* [21] found evidence of disease in only ∼11% of then extinct, extinct in the wild, and critically endangered amphibians reported by the Red List as threatened by a pathogen or parasite (Fig. 1). Re-analysis of the Red List four years later indicates that little progress has been made to confirm the causal role of disease, specifically chytridiomycosis, in the global loss of amphibians. We present these new findings here and discuss the implications for amphibian conservation.

## Methods

We repeated the analyses of Smith et al. 2006 [21], individually examining the “full account” of amphibian species assessments in the 2010 Red List to determine those threatened by infectious disease, specifically chytridiomycosis and/or the causal agent *Bd*. Smith et al. 's 2006 [21] data included amphibians threatened generally by disease (the authors did not distinguish those threatened by chytridiomycosis, though the pathogen was the likely cause). Data compiled for this study includes only amphibian species specifically threatened by chytridiomycosis/*Bd* but these constitute 97% of all disease-threatened amphibian species listed in the Red List. Whereas Smith et al. [21] only examined species extinct, extinct in the wild and critically endangered, we examined these status categories as well as endangered and vulnerable.

Data was compiled from the Red List in June–August 2010 resulting in 432 amphibian species with disease reported as a threat in the full account. Eleven species had pathogens other than *Bd* listed as the disease agent of concern or reported the threat of a ‘disease’ but did not specify which. These were excluded from analyses (CR: *Rhinella amabilis*, *Ecnomiohyla rabborum*, *Ambystoma mexicanum*; EN: *Atelognathus patagonicus*, *Atelopus oxapampae*; VU: *Batrachuperus pinchonii*, *Prestimantis schultei*, *Rana draytonii*, *Rana latastei*, *Cochranella punctulata*, *Rhinella quechua*.) The 421 remaining amphibian species assessments explicitly listed threat from the disease chytridiomycosis or the pathogen *Bd* and were therefore the basis for our analyses (Table S1).

Each species account was read in full and species assigned to one of two categories based on level of evidence in support of a chytridiomycosis/*Bd* threat:

1. Evidence for Chytridiomycosis: chytridiomycosis is a confirmed threat and/or the fungus *Bd* has been identified in at least one of the species' populations where it is confirmed to cause disease.
2. Chytridiomycosis Hypothesized: no evidence exists to support chytridiomycosis as a threat, *Bd* has not been detected in any of the species' populations, *Bd* has been hypothesized to threaten the species in the future, or the species is determined asymptomatic when infected.

We determined the proportion of amphibian species that deteriorated in Red List status by three or more categories from 1980–2004 that are purportedly threatened by chytridiomycosis/*Bd*. Amphibian species that deteriorated in status by more than three categories were identified using data collected by Hoffmann et al. [4] (Table S2). We focused on amphibian species that deteriorated by three or more categories because they are considered the most threatened and are likely to have garnered significant attention from the scientific community. For each amphibian species, we also examined whether there was evidence confirming the purported threat of chytridiomycosis/*Bd*.

We utilized the 2010 Red List for our analyses, however, data on several species had not been updated since 2004, increasing the potential for a significant discrepancy between real-time science (specifically publications after 2004) and Red List assessment status. To test for this, we conducted advanced literature surveys of the 123 amphibian species last assessed in 2004 as critically endangered and hypothesized to be threatened by chytridiomycosis/*Bd* to determine if evidence for the disease (as above) existed in the primary literature but were not yet incorporated into Red List assessments (Table S3). We searched for published primary literature in Google Scholar (validated against Web of Science and PubMed) using the following search strings: scientific name chytridiomycosis, common name chytridiomycosis, scientific name *Batrachochytrium dendrobatidis*, common name *Batrachochytrium dendrobatidis*. Each article returned was read in full to identify inconsistencies with evidence level reported for each of the 123 species hypothesized by the Red List to be threatened by chytridiomycosis/*Bd*.

## Results

In 2006, Smith et al. [21] found evidence of disease in only ∼11% of then extinct, extinct in the wild, and critically endangered amphibians reported by the Red List as threatened by a pathogen or parasite (Fig. 1). Re-analysis of the Red List four years later indicates that little progress has been made to confirm the causal role of disease, specifically chytridiomycosis, in the global loss of amphibians (Fig. 1). Of 421 Red List amphibian species purportedly threatened by chytridiomycosis, evidence that *Bd* has been identified in the species and confirmed to cause clinical disease exists for only fifteen percent (Fig. 1). Of the 36 amphibian species reported by Hoffmann *et al.* to have deteriorated by three or more Red List categories from 1980–2004, the majority are purportedly threatened by chytridiomycosis (28 species), but only 39% of these are backed by scientific evidence (Table S2). Advanced primary literature surveys on the 123 critically endangered amphibian species last assessed by the Red List in 2004 identified 751 Google Scholar search results concerning these species (Table S3). Within these results we found evidence confirming *Bd* infections for only ten species.

## Discussion

The lack of scientific evidence to support the listing of chytridiomycosis as a threat to 358 Red Listed amphibians (Fig. 1; Table S1) is surprising given the magnitude of attention the disease received in the last decade. Analyses comparing Red List assessments with the primary literature rule out major discrepancies with real-time science. Of the 123 hypothesized chytridiomycosis-threatened critically endangered amphibians reviewed, only ten had confirmed *Bd* infections reported in the primary literature. Of these only five had evidence that the fungus ultimately caused chytridiomycosis (Table S3). This is consistent with a growing body of research suggesting that many species harbor *Bd* but never develop chytridiomycosis or experience resulting population declines (i.e. the North American bullfrog *Rana catesbeiana*) [11], [22]–[24]. The high proportion of Red List amphibians with a purported chytridiomycosis threat not backed by scientific evidence implies a strong precautionary approach adopted by assessment teams. Erring on the side of caution can be critical to conservation and so Red List assessments often include hypothesized or future threats deemed likely to have irreversible effects [21], [25]. As was the finding of Smith et al. [21], precaution continues to drive the listing of chytridiomycosis as a threat to many Red Listed amphibians. The precautionary principle is valid approach when dealing with the world's most threatened vertebrate taxa, but focused scientific efforts to distinguish between real and hypothesized threats should be a larger priority.

In the four and a half years since Smith et al. 's [21] analyses, $4,119,851 in awarded NSF grants, £1,617,371 in awarded UK Research Council grants, and 249 publications have been dedicated to chytridiomycosis research [26], [27]. In addition, the Office Internationale des Epizooties (OIE) recognized chytridiomycosis as a reportable threat to wildlife, the Amphibian Survival Alliance was formed, and the U.S. Pet Industry Joint Advisory Council implemented their *Bd*-Free ‘Phibs campaign. Despite a wealth of science, policy and industry efforts like these, our findings reveal that little progress has been made to acquire science-based evidence on the role of chytridiomycosis in species-specific amphibian declines. The recent IUCN Amphibian Conservation Action Plan (ACAP) should help to fill this knowledge gap, calling for an ambitious US $25 million 5-year chytridiomycosis research agenda with studies targeted to 1) sites where amphibians are undergoing enigmatic declines due to the disease, and 2) sites where *Bd* is present, yet populations of amphibians persist without declines [28]. If chytridiomycosis is as serious a threat as ACAP suggests, and most experts believe, knowing which species it will harm is equally critical to prioritizing amphibian conservation efforts. Determining this for each of the ∼350 species hypothesized to be threatened by the disease, or predicated to be in the future, is unrealistic. However, a step in the right direction would be prioritizing select species with a hypothesized chytridiomycosis threat for in situ studies that provide evidence of disease presence, or rule it out. The success of broad surveys like this would depend on cost effect and rapid diagnostic techniques. Such studies, especially those focused on qPCR in preserved and field collected specimens, have already proven successful in accumulating species-specific data on *Bd* presence and chytridiomycosis effects at the population scale [11], [29]–[34]. Support to continue this level of research would go a long way toward shoring up the science behind Red List assessments and subsequently prioritize amphibian conservation.

## Supporting Information

Table S1 Species examined in the Red List for evidence of chytridiomycosis. “STATUS” indicates Red List abbreviations: CR, Critically Endangered; EN, Endangered; EW, Extinct in the Wild; EX, Extinct; Vul, Vulnerable.
(DOC)

Table S2 Amphibian species that deteriorated in IUCN Red List status by more than three categories from 1980–2004 examined for evidence of chytridiomycosis. N/A implies that other threat besides chytridiomycosis was causal factor of decline (e.g. hunting or land use change).
(DOC)

Table S3 Critically endangered species used in advanced literature surveys to investigate lags between Red List assessments and the primarily scientific literature. Discrepancies existed for only 10 of 123 species. In all cases the Red List hypothesized the threat of chytridiomycosis whereas primary sources documented the presence of *Bd* in wild populations (although only five manifested in clinical disease).
(DOC)
