# Supplementary material for: Examining the Evidence for Chytridiomycosis in Threatened Amphibian Species
Source: PLoS One. 2011 Aug 3;6(8):e23150. doi: 10.1371/journal.pone.0023150 (PMC3149636; doi:10.1371/journal.pone.0023150)
Supplement: Table S1 — Species examined in the Red List for evidence of chytridiomycosis. “STATUS” indicates Red List abbreviations: CR, Critically Endangered; EN, Endangered; EW, Extinct in the Wild; EX, Extinct; Vul, Vulnerable. (DOC) [file pone.0023150.s001.doc]

**Table S1**

| **GENUS** | **SPECIES** | **STATUS** | **CHYTRID EVIDENCE** |
| --- | --- | --- | --- |
| *Agalychnis* | *annae* | EN | Hypothesized |
| *Agalychnis* | *moreletii* | CR | Hypothesized |
| *Allobates* | *olfersioides* | VU | Evidence |
| *Alytes* | *dickhilleni* | VU | Hypothesized |
| *Alytes* | *muletensis* | VU | Evidence |
| *Anaxyrus* | *baxteri* | EW | Evidence |
| *Anaxyrus* | *canorus* | EN | Evidence |
| *Aromobates* | *alboguttatus* | EN | Hypothesized |
| *Aromobates* | *leopardalis* | CR | Hypothesized |
| *Aromobates* | *nocturnus* | CR | Hypothesized |
| *Atelopus* | *andinus* | CR | Hypothesized |
| *Atelopus* | *angelito* | CR | Hypothesized |
| *Atelopus* | *arsyecue* | CR | Hypothesized |
| *Atelopus* | *arthuri* | CR | Hypothesized |
| *Atelopus* | *balios* | CR | Hypothesized |
| *Atelopus* | *bomolochos* | CR | Evidence |
| *Atelopus* | *boulengeri* | CR | Hypothesized |
| *Atelopus* | *carauta* | CR | Hypothesized |
| *Atelopus* | *carbonerensis* | CR | Evidence |
| *Atelopus* | *carrikeri* | CR | Hypothesized |
| *Atelopus* | *certus* | EN | Hypothesized |
| *Atelopus* | *chiriquiensis* | CR | Evidence |
| *Atelopus* | *chocoensis* | CR | Hypothesized |
| *Atelopus* | *chrysocorallus* | CR | Hypothesized |
| *Atelopus* | *coynei* | CR | Hypothesized |
| *Atelopus* | *cruciger* | CR | Evidence |
| *Atelopus* | *dimorphus* | EN | Hypothesized |
| *Atelopus* | *ebenoides* | CR | Hypothesized |
| *Atelopus* | *elegans* | CR | Hypothesized |
| *Atelopus* | *epikeisthos* | CR | Hypothesized |
| *Atelopus* | *erythropus* | CR | Hypothesized |
| *Atelopus* | *eusebianus* | CR | Hypothesized |
| *Atelopus* | *exiguus* | CR | Hypothesized |
| *Atelopus* | *famelicus* | CR | Hypothesized |
| *Atelopus* | *farci* | CR | Hypothesized |
| *Atelopus* | *flavescens* | VU | Hypothesized |
| *Atelopus* | *franciscus* | VU | Hypothesized |
| *Atelopus* | *galactogaster* | CR | Hypothesized |
| *Atelopus* | *glyphus* | CR | Hypothesized |
| *Atelopus* | *guanujo* | CR | Hypothesized |
| *Atelopus* | *guitarraensis* | CR | Hypothesized |
| *Atelopus* | *halihelos* | CR | Hypothesized |
| *Atelopus* | *ignescens* | EX | Hypothesized |
| *Atelopus* | *laetissimus* | CR | Hypothesized |
| *Atelopus* | *limosus* | EN | Hypothesized |
| *Atelopus* | *longibrachius* | EN | Hypothesized |
| *Atelopus* | *longirostris* | EX | Hypothesized |
| *Atelopus* | *lozanoi* | CR | Hypothesized |
| *Atelopus* | *lynchi* | CR | Hypothesized |
| *Atelopus* | *mandingues* | CR | Hypothesized |
| *Atelopus* | *mindoensis* | CR | Hypothesized |
| *Atelopus* | *minutulus* | CR | Hypothesized |
| *Atelopus* | *mittermeieri* | EN | Evidence |
| *Atelopus* | *monohernandezii* | CR | Hypothesized |
| *Atelopus* | *mucubajiensis* | CR | Evidence |
| *Atelopus* | *muisca* | CR | Hypothesized |
| *Atelopus* | *nahumae* | CR | Hypothesized |
| *Atelopus* | *nanay* | CR | Hypothesized |
| *Atelopus* | *nepiozomus* | CR | Hypothesized |
| *Atelopus* | *nicefori* | CR | Hypothesized |
| *Atelopus* | *onorei* | CR | Hypothesized |
| *Atelopus* | *oxyrhynchus* | CR | Hypothesized |
| *Atelopus* | *pachydermus* | CR | Hypothesized |
| *Atelopus* | *pedimarmoratus* | CR | Hypothesized |
| *Atelopus* | *peruensis* | CR | Hypothesized |
| *Atelopus* | *petersi* | CR | Hypothesized |
| *Atelopus* | *petriruizi* | CR | Hypothesized |
| *Atelopus* | *pictiventris* | CR | Hypothesized |
| *Atelopus* | *pinangoi* | CR | Hypothesized |
| *Atelopus* | *planispina* | CR | Hypothesized |
| *Atelopus* | *pulcher* | CR | Evidence |
| *Atelopus* | *pyrodactylus* | CR | Hypothesized |
| *Atelopus* | *quimbaya* | CR | Hypothesized |
| *Atelopus* | *reticulatus* | CR | Hypothesized |
| *Atelopus* | *seminiferus* | CR | Hypothesized |
| *Atelopus* | *senex* | CR | Hypothesized |
| *Atelopus* | *sernai* | CR | Hypothesized |
| *Atelopus* | *simulatus* | CR | Hypothesized |
| *Atelopus* | *sonsonensis* | CR | Hypothesized |
| *Atelopus* | *sorianoi* | CR | Evidence |
| *Atelopus* | *spumarius* | VU | Hypothesized |
| *Atelopus* | *spurrelli* | VU | Hypothesized |
| *Atelopus* | *subornatus* | CR | Hypothesized |
| *Atelopus* | *tamaensis* | CR | Hypothesized |
| *Atelopus* | *tricolor* | VU | Hypothesized |
| *Atelopus* | *varius* | CR | Evidence |
| *Atelopus* | *walkeri* | CR | Hypothesized |
| *Atelopus* | *zeteki* | CR | Hypothesized |
| *Bolitoglossa* | *conanti* | EN | Evidence |
| *Bolitoglossa* | *diaphora* | CR | Evidence |
| *Bolitoglossa* | *magnifica* | EN | Hypothesized |
| *Bolitoglossa* | *pesrubra* | VU | Hypothesized |
| *Bolitoglossa* | *sombra* | VU | Hypothesized |
| *Bolitoglossa* | *sooyorum* | EN | Hypothesized |
| *Bolitoglossa* | *subpalmata* | EN | Hypothesized |
| *Bromeliohyla* | *bromeliacia* | EN | Hypothesized |
| *Bromeliohyla* | *dendroscarta* | CR | Hypothesized |
| *Centrolene* | *audax* | EN | Hypothesized |
| *Centrolene* | *azulae* | EN | Hypothesized |
| *Centrolene* | *ballux* | CR | Hypothesized |
| *Centrolene* | *buckleyi* | VU | Hypothesized |
| *Centrolene* | *geckoideum* | VU | Hypothesized |
| *Centrolene* | *gemmatum* | CR | Hypothesized |
| *Centrolene* | *heloderma* | CR | Hypothesized |
| *Centrolene* | *hesperium* | VU | Hypothesized |
| *Centrolene* | *lynchi* | EN | Hypothesized |
| *Centrolene* | *peristictum* | VU | Hypothesized |
| *Centrolene* | *pipilatum* | EN | Hypothesized |
| *Charadrahyla* | *altipotens* | CR | Hypothesized |
| *Charadrahyla* | *nephila* | VU | Hypothesized |
| *Charadrahyla* | *trux* | CR | Hypothesized |
| *Chiropterotriton* | *cracens* | EN | Hypothesized |
| *Chiropterotriton* | *multidentatus* | EN | Hypothesized |
| *Cochranella* | *saxiscandens* | EN | Hypothesized |
| *Craugastor* | *anciano* | CR | Hypothesized |
| *Craugastor* | *andi* | CR | Hypothesized |
| *Craugastor* | *angelicus* | CR | Hypothesized |
| *Craugastor* | *aurilegulus* | EN | Evidence |
| *Craugastor* | *azueroensis* | EN | Hypothesized |
| *Craugastor* | *brocchi* | VU | Hypothesized |
| *Craugastor* | *catalinae* | CR | Hypothesized |
| *Craugastor* | *charadra* | EN | Hypothesized |
| *Craugastor* | *chrysozetetes* | EX | Hypothesized |
| *Craugastor* | *cruzi* | CR | Hypothesized |
| *Craugastor* | *daryi* | EN | Hypothesized |
| *Craugastor* | *emcelae* | CR | Hypothesized |
| *Craugastor* | *emleni* | CR | Hypothesized |
| *Craugastor* | *epochthidius* | CR | Hypothesized |
| *Craugastor* | *escoces* | EX | Hypothesized |
| *Craugastor* | *fecundus* | CR | Hypothesized |
| *Craugastor* | *fleischmanni* | CR | Hypothesized |
| *Craugastor* | *greggi* | CR | Hypothesized |
| *Craugastor* | *guerreroensis* | CR | Hypothesized |
| *Craugastor* | *inachus* | EN | Hypothesized |
| *Craugastor* | *laevissimus* | EN | Hypothesized |
| *Craugastor* | *lineatus* | CR | Hypothesized |
| *Craugastor* | *merendonensis* | CR | Hypothesized |
| *Craugastor* | *milesi* | CR | Hypothesized |
| *Craugastor* | *obesus* | EN | Hypothesized |
| *Craugastor* | *olanchano* | CR | Hypothesized |
| *Craugastor* | *omoaensis* | CR | Hypothesized |
| *Craugastor* | *pechorum* | EN | Hypothesized |
| *Craugastor* | *polymniae* | CR | Hypothesized |
| *Craugastor* | *punctariolus* | EN | Hypothesized |
| *Craugastor* | *ranoides* | CR | Hypothesized |
| *Craugastor* | *ranoides* | EN | Hypothesized |
| *Craugastor* | *sabrinus* | EN | Hypothesized |
| *Craugastor* | *saltuarius* | CR | Hypothesized |
| *Craugastor* | *sandersoni* | EN | Hypothesized |
| *Craugastor* | *stadelmani* | CR | Hypothesized |
| *Craugastor* | *tabasarae* | CR | Evidence |
| *Craugastor* | *taurus* | CR | Hypothesized |
| *Craugastor* | *trachydermus* | CR | Hypothesized |
| *Cryptotriton* | *monzoni* | CR | Hypothesized |
| *Duellmanohyla* | *chamulae* | EN | Hypothesized |
| *Duellmanohyla* | *ignicolor* | EN | Hypothesized |
| *Duellmanohyla* | *lythrodes* | EN | Hypothesized |
| *Duellmanohyla* | *salvavida* | CR | Hypothesized |
| *Duellmanohyla* | *schmidtorum* | VU | Hypothesized |
| *Duellmanohyla* | *soralia* | CR | Evidence |
| *Duellmanohyla* | *uranochroa* | CR | Evidence |
| *Ecnomiohyla* | *echinata* | CR | Hypothesized |
| *Eleutherodactylus* | *barlagnei* | EN | Hypothesized |
| *Eleutherodactylus* | *cooki* | VU | Hypothesized |
| *Eleutherodactylus* | *eneidae* | CR | Hypothesized |
| *Eleutherodactylus* | *gryllus* | EN | Hypothesized |
| *Eleutherodactylus* | *hedricki* | EN | Hypothesized |
| *Eleutherodactylus* | *jasperi* | CR | Hypothesized |
| *Eleutherodactylus* | *karlschmidti* | CR | Hypothesized |
| *Eleutherodactylus* | *locustus* | CR | Hypothesized |
| *Eleutherodactylus* | *longipes* | VU | Hypothesized |
| *Eleutherodactylus* | *orcutti* | CR | Hypothesized |
| *Eleutherodactylus* | *patriciae* | EN | Evidence |
| *Eleutherodactylus* | *pituinus* | EN | Evidence |
| *Eleutherodactylus* | *portoricensis* | EN | Evidence |
| *Eleutherodactylus* | *richmondi* | CR | Hypothesized |
| *Eleutherodactylus* | *ruthae* | EN | Hypothesized |
| *Eleutherodactylus* | *schmidti* | CR | Hypothesized |
| *Eleutherodactylus* | *semipalmatus* | CR | Hypothesized |
| *Eleutherodactylus* | *symingtoni* | CR | Hypothesized |
| *Eleutherodactylus* | *turquinensis* | CR | Hypothesized |
| *Eleutherodactylus* | *unicolor* | VU | Hypothesized |
| *Eleutherodactylus* | *wightmanae* | EN | Hypothesized |
| *Epipedobates* | *tricolor* | EN | Hypothesized |
| *Euproctus* | *platycephalus* | EN | Evidence |
| *Exerodonta* | *juanitae* | VU | Hypothesized |
| *Exerodonta* | *melanomma* | VU | Hypothesized |
| *Exerodonta* | *perkinsi* | CR | Hypothesized |
| *Exerodonta* | *pinorum* | VU | Hypothesized |
| *Gastrotheca* | *cornuta* | EN | Evidence |
| *Gastrotheca* | *dendronastes* | VU | Hypothesized |
| *Gastrotheca* | *guentheri* | VU | Hypothesized |
| *Gastrotheca* | *litonedis* | EN | Hypothesized |
| *Gastrotheca* | *orophylax* | EN | Hypothesized |
| *Gastrotheca* | *ovifera* | EN | Hypothesized |
| *Gastrotheca* | *plumbea* | VU | Hypothesized |
| *Gastrotheca* | *pseustes* | EN | Evidence |
| *Gastrotheca* | *riobambae* | EN | Hypothesized |
| *Gastrotheca* | *splendens* | EN | Hypothesized |
| *Heleioporus* | *australiacus* | VU | Evidence |
| *Hyalinobatrachium* | *guairarepanense* | EN | Hypothesized |
| *Hyla* | *bocourti* | CR | Hypothesized |
| *Hylomantis* | *lemur* | CR | Hypothesized |
| *Hyloscirtus* | *colymba* | CR | Hypothesized |
| *Hyloscirtus* | *lindae* | VU | Hypothesized |
| *Hyloscirtus* | *pantostictus* | EN | Hypothesized |
| *Hyloscirtus* | *platydactylus* | VU | Hypothesized |
| *Hyloscirtus* | *psarolaimus* | EN | Hypothesized |
| *Hyloscirtus* | *ptychodactylus* | CR | Hypothesized |
| *Hyloscirtus* | *staufferorum* | EN | Hypothesized |
| *Hyloscirtus* | *torrenticola* | VU | Hypothesized |
| *Hyloxalus* | *anthracinus* | CR | Hypothesized |
| *Hyloxalus* | *delatorreae* | CR | Hypothesized |
| *Hyloxalus* | *elachyhistus* | EN | Hypothesized |
| *Hyloxalus* | *pulchellus* | VU | Hypothesized |
| *Hyloxalus* | *vertebralis* | CR | Hypothesized |
| *Hypodactylus* | *dolops* | VU | Hypothesized |
| *Hypsiboas* | *cymbalum* | CR | Hypothesized |
| *Incilius* | *cycladen* | VU | Hypothesized |
| *Incilius* | *fastidiosus* | CR | Hypothesized |
| *Incilius* | *holdridgei* | EX | Hypothesized |
| *Incilius* | *ibarrai* | EN | Hypothesized |
| *Incilius* | *periglenes* | EX | Hypothesized |
| *Incilius* | *peripatetes* | CR | Hypothesized |
| *Incilius* | *tacanensis* | EN | Hypothesized |
| *Incilius* | *tutelarius* | EN | Hypothesized |
| *Isthmohyla* | *angustilineata* | CR | Hypothesized |
| *Isthmohyla* | *calypsa* | CR | Hypothesized |
| *Isthmohyla* | *debilis* | CR | Hypothesized |
| *Isthmohyla* | *graceae* | CR | Evidence |
| *Isthmohyla* | *pictipes* | EN | Hypothesized |
| *Isthmohyla* | *rivularis* | CR | Hypothesized |
| *Isthmohyla* | *tica* | CR | Hypothesized |
| *Leiopelma* | *archeyi* | CR | Evidence |
| *Leiopelma* | *hamiltoni* | EN | Hypothesized |
| *Leiopelma* | *hochstetteri* | VU | Hypothesized |
| *Leiopelma* | *pakeka* | VU | Hypothesized |
| *Leptodactylus* | *fallax* | CR | Evidence |
| *Leptophryne* | *cruentata* | CR | Hypothesized |
| *Lithobates* | *chiricahuensis* | VU | Evidence |
| *Lithobates* | *omiltemanus* | CR | Hypothesized |
| *Lithobates* | *sevosus* | CR | Evidence |
| *Lithobates* | *sierramadrensis* | VU | Hypothesized |
| *Lithobates* | *subaquavocalis* | CR | Evidence |
| *Lithobates* | *tarahumarae* | VU | Evidence |
| *Lithobates* | *vibicarius* | CR | Evidence |
| *Litoria* | *aurea* | VU | Evidence |
| *Litoria* | *becki* | VU | Hypothesized |
| *Litoria* | *booroolongensis* | CR | Hypothesized |
| *Litoria* | *castanea* | CR | Hypothesized |
| *Litoria* | *dayi* | EN | Evidence |
| *Litoria* | *lorica* | CR | Hypothesized |
| *Litoria* | *myola* | CR | Hypothesized |
| *Litoria* | *nannotis* | EN | Hypothesized |
| *Litoria* | *nyakalensis* | CR | Hypothesized |
| *Litoria* | *piperata* | CR | Hypothesized |
| *Litoria* | *raniformis* | EN | Evidence |
| *Litoria* | *rheocola* | EN | Evidence |
| *Litoria* | *spenceri* | CR | Evidence |
| *Mannophryne* | *caquetio* | CR | Hypothesized |
| *Mannophryne* | *cordilleriana* | CR | Evidence |
| *Mannophryne* | *lamarcai* | CR | Hypothesized |
| *Mannophryne* | *neblina* | CR | Hypothesized |
| *Mannophryne* | *olmonae* | CR | Hypothesized |
| *Mannophryne* | *riveroi* | EN | Hypothesized |
| *Megastomatohyla* | *pellita* | CR | Hypothesized |
| *Mixophyes* | *balbus* | VU | Hypothesized |
| *Mixophyes* | *fleayi* | EN | Evidence |
| *Nectophrynoides* | *asperginis* | EW | Evidence |
| *Neurergus* | *microspilotus* | EN | Hypothesized |
| *Nymphargus* | *griffithsi* | VU | Hypothesized |
| *Nymphargus* | *megacheirus* | EN | Hypothesized |
| *Oedipina* | *poelzi* | EN | Hypothesized |
| *Oophaga* | *arborea* | EN | Hypothesized |
| *Osteopilus* | *pulchrilineatus* | EN | Hypothesized |
| *Osteopilus* | *vastus* | EN | Evidence |
| *Petropedetes* | *dutoiti* | CR | Hypothesized |
| *Petropedetes* | *martiensseni* | EN | Hypothesized |
| *Petropedetes* | *yakusini* | EN | Evidence |
| *Philoria* | *frosti* | CR | Hypothesized |
| *Phyllomedusa* | *ecuatoriana* | EN | Hypothesized |
| *Plectrohyla* | *acanthodes* | CR | Hypothesized |
| *Plectrohyla* | *arborescandens* | EN | Hypothesized |
| *Plectrohyla* | *avia* | CR | Hypothesized |
| *Plectrohyla* | *calthula* | CR | Hypothesized |
| *Plectrohyla* | *calvicollina* | CR | Hypothesized |
| *Plectrohyla* | *celata* | CR | Hypothesized |
| *Plectrohyla* | *cembra* | CR | Hypothesized |
| *Plectrohyla* | *charadricola* | EN | Hypothesized |
| *Plectrohyla* | *chryses* | CR | Hypothesized |
| *Plectrohyla* | *chrysopleura* | CR | Evidence |
| *Plectrohyla* | *crassa* | CR | Hypothesized |
| *Plectrohyla* | *cyanomma* | CR | Hypothesized |
| *Plectrohyla* | *cyclada* | EN | Hypothesized |
| *Plectrohyla* | *dasypus* | CR | Evidence |
| *Plectrohyla* | *ephemera* | CR | Hypothesized |
| *Plectrohyla* | *exquisita* | CR | Evidence |
| *Plectrohyla* | *glandulosa* | EN | Hypothesized |
| *Plectrohyla* | *guatemalensis* | CR | Hypothesized |
| *Plectrohyla* | *hartwegi* | CR | Hypothesized |
| *Plectrohyla* | *hazelae* | CR | Hypothesized |
| *Plectrohyla* | *ixil* | CR | Hypothesized |
| *Plectrohyla* | *lacertosa* | EN | Hypothesized |
| *Plectrohyla* | *matudai* | VU | Hypothesized |
| *Plectrohyla* | *mykter* | EN | Hypothesized |
| *Plectrohyla* | *pachyderma* | CR | Hypothesized |
| *Plectrohyla* | *pentheter* | EN | Hypothesized |
| *Plectrohyla* | *pokomchi* | CR | Hypothesized |
| *Plectrohyla* | *psarosema* | CR | Hypothesized |
| *Plectrohyla* | *psiloderma* | EN | Hypothesized |
| *Plectrohyla* | *pycnochila* | CR | Hypothesized |
| *Plectrohyla* | *quecchi* | CR | Hypothesized |
| *Plectrohyla* | *robertsorum* | EN | Hypothesized |
| *Plectrohyla* | *sabrina* | CR | Hypothesized |
| *Plectrohyla* | *sagorum* | EN | Hypothesized |
| *Plectrohyla* | *siopela* | CR | Hypothesized |
| *Plectrohyla* | *tecunumani* | CR | Hypothesized |
| *Plectrohyla* | *teuchestes* | CR | Hypothesized |
| *Plectrohyla* | *thorectes* | CR | Hypothesized |
| *Pristimantis* | *albericoi* | CR | Hypothesized |
| *Pristimantis* | *bicolor* | VU | Hypothesized |
| *Pristimantis* | *calcarulatus* | VU | Hypothesized |
| *Pristimantis* | *cremnobates* | EN | Hypothesized |
| *Pristimantis* | *crenunguis* | EN | Hypothesized |
| *Pristimantis* | *crucifer* | VU | Hypothesized |
| *Pristimantis* | *diaphonus* | VU | Hypothesized |
| *Pristimantis* | *diogenes* | VU | Hypothesized |
| *Pristimantis* | *duellmani* | VU | Hypothesized |
| *Pristimantis* | *fallax* | EN | Hypothesized |
| *Pristimantis* | *fetosus* | EN | Hypothesized |
| *Pristimantis* | *ginesi* | EN | Hypothesized |
| *Pristimantis* | *gracilis* | VU | Hypothesized |
| *Pristimantis* | *ignicolor* | EN | Hypothesized |
| *Pristimantis* | *incanus* | EN | Hypothesized |
| *Pristimantis* | *jorgevelosai* | EN | Hypothesized |
| *Pristimantis* | *lancinii* | EN | Hypothesized |
| *Pristimantis* | *lichenoides* | CR | Hypothesized |
| *Pristimantis* | *nigrogriseus* | VU | Hypothesized |
| *Pristimantis* | *penelopus* | VU | Hypothesized |
| *Pristimantis* | *prolatus* | EN | Hypothesized |
| *Pristimantis* | *quinquagesimus* | VU | Hypothesized |
| *Pristimantis* | *ruedai* | VU | Hypothesized |
| *Pristimantis* | *scoloblepharus* | EN | Hypothesized |
| *Pristimantis* | *scolodiscus* | EN | Hypothesized |
| *Pristimantis* | *signifer* | VU | Hypothesized |
| *Pristimantis* | *sulculus* | EN | Hypothesized |
| *Pristimantis* | *urichi* | EN | Hypothesized |
| *Pristimantis* | *verecundus* | VU | Hypothesized |
| *Pristimantis* | *vertebralis* | VU | Hypothesized |
| *Pristimantis* | *zophus* | EN | Hypothesized |
| *Prostherapis* | *dunni* | CR | Hypothesized |
| *Pseudoeurycea* | *unguidentis* | CR | Hypothesized |
| *Pseudophryne* | *corroboree* | CR | Evidence |
| *Pseudophryne* | *pengilleyi* | EN | Evidence |
| *Ptychohyla* | *dendrophasma* | CR | Hypothesized |
| *Ptychohyla* | *erythromma* | EN | Hypothesized |
| *Ptychohyla* | *hypomykter* | CR | Evidence |
| *Ptychohyla* | *legleri* | EN | Hypothesized |
| *Ptychohyla* | *leonhardschultzei* | EN | Hypothesized |
| *Ptychohyla* | *macrotympanum* | CR | Hypothesized |
| *Ptychohyla* | *panchoi* | EN | Hypothesized |
| *Ptychohyla* | *salvadorensis* | EN | Hypothesized |
| *Ptychohyla* | *sanctaecrucis* | CR | Hypothesized |
| *Ptychohyla* | *spinipollex* | EN | Hypothesized |
| *Rana* | *muscosa* | EN | Evidence |
| *Rana* | *sierrae* | EN | Evidence |
| *Ranitomeya* | *abdita* | CR | Hypothesized |
| *Rheobatrachus* | *silus* | EX | Hypothesized |
| *Rheobatrachus* | *vitellinus* | EX | Hypothesized |
| *Rhinella* | *chrysophora* | EN | Hypothesized |
| *Rhinoderma* | *darwinii* | VU | Hypothesized |
| *Rhinoderma* | *rufum* | CR | Hypothesized |
| *Strabomantis* | *cheiroplethus* | VU | Hypothesized |
| *Strabomantis* | *cornutus* | VU | Hypothesized |
| *Strabomantis* | *necerus* | VU | Hypothesized |
| *Taudactylus* | *acutirostris* | CR | Evidence |
| *Taudactylus* | *diurnus* | EX | Hypothesized |
| *Taudactylus* | *eungellensis* | CR | Evidence |
| *Taudactylus* | *pleione* | CR | Hypothesized |
| *Taudactylus* | *rheophilus* | CR | Hypothesized |
| *Telmatobius* | *arequipensis* | VU | Hypothesized |
| *Telmatobius* | *atacamensis* | CR | Hypothesized |
| *Telmatobius* | *brevipes* | EN | Hypothesized |
| *Telmatobius* | *brevirostris* | EN | Hypothesized |
| *Telmatobius* | *carrillae* | VU | Hypothesized |
| *Telmatobius* | *ceiorum* | EN | Hypothesized |
| *Telmatobius* | *cirrhacelis* | CR | Hypothesized |
| *Telmatobius* | *colanensis* | EN | Hypothesized |
| *Telmatobius* | *culeus* | CR | Hypothesized |
| *Telmatobius* | *degener* | EN | Hypothesized |
| *Telmatobius* | *edaphonastes* | EN | Hypothesized |
| *Telmatobius* | *espadai* | CR | Evidence |
| *Telmatobius* | *gigas* | CR | Hypothesized |
| *Telmatobius* | *hauthali* | VU | Hypothesized |
| *Telmatobius* | *hintoni* | VU | Hypothesized |
| *Telmatobius* | *hockingi* | VU | Hypothesized |
| *Telmatobius* | *hypselocephalus* | EN | Hypothesized |
| *Telmatobius* | *ignavus* | EN | Hypothesized |
| *Telmatobius* | *laticeps* | EN | Hypothesized |
| *Telmatobius* | *latirostris* | EN | Hypothesized |
| *Telmatobius* | *marmoratus* | VU | Evidence |
| *Telmatobius* | *mayoloi* | EN | Hypothesized |
| *Telmatobius* | *necopinus* | EN | Hypothesized |
| *Telmatobius* | *niger* | CR | Evidence |
| *Telmatobius* | *oxycephalus* | VU | Hypothesized |
| *Telmatobius* | *pefauri* | CR | Hypothesized |
| *Telmatobius* | *peruvianus* | VU | Hypothesized |
| *Telmatobius* | *pisanoi* | EN | Hypothesized |
| *Telmatobius* | *platycephalus* | EN | Hypothesized |
| *Telmatobius* | *schreiteri* | EN | Hypothesized |
| *Telmatobius* | *scrocchii* | EN | Hypothesized |
| *Telmatobius* | *sibiricus* | EN | Hypothesized |
| *Telmatobius* | *stephani* | EN | Hypothesized |
| *Telmatobius* | *thompsoni* | EN | Hypothesized |
| *Telmatobius* | *truebae* | EN | Hypothesized |
| *Telmatobius* | *vellardi* | CR | Hypothesized |
| *Telmatobius* | *verrucosus* | VU | Hypothesized |
| *Telmatobius* | *yuracare* | VU | Hypothesized |
| *Telmatobius* | *zapahuirensis* | CR | Hypothesized |
| *Thoropa* | *lutzi* | EN | Hypothesized |
| *Thoropa* | *petropolitana* | VU | Hypothesized |
