# Supplementary material for: Examining the Evidence for Chytridiomycosis in Threatened Amphibian Species
Source: PLoS One. 2011 Aug 3;6(8):e23150. doi: 10.1371/journal.pone.0023150 (PMC3149636; doi:10.1371/journal.pone.0023150)
Supplement: Table S2 — Amphibian species that deteriorated in IUCN Red List status by more than three categories from 1980–2004 examined for evidence of chytridiomycosis. N/A implies that other threat besides chytridiomycosis was causal factor of decline (e.g. hunting or land use change). (DOC) [file pone.0023150.s002.doc]

**Table S2**

| **Species** | **# IUCN Cat. Decreased** | **Chytrid Evidence** |
| --- | --- | --- |
| *Agalychnis moreletii* | 4 | Hypothesized |
| *Andrias davidianus* | 3 | N/A |
| *Argenteohyla siemersi* | 3 | N/A |
| *Atelopus andinus* | 3 | Hypothesized |
| *Atelopus carbonerensis* | 3 | Evidence |
| *Atelopus chrysocorallus* | 3 | Hypothesized |
| *Atelopus ignescens* | 3 | Evidence |
| *Atelopus mucubajiensis* | 3 | Evidence |
| *Atelopus muisca* | 3 | Hypothesized |
| *Atelopus nanay* | 3 | Hypothesized |
| *Atelopus peruensis* | 3 | Hypothesized |
| *Atelopus varius* | 3 | Evidence |
| *Incilius holdridgei* | 3 | Hypothesized |
| *Incilius periglenes* | 3 | Evidence |
| *Centrolene heloderma* | 3 | Hypothesized |
| *Hyloxalus vertebralis* | 3 | Hypothesized |
| *Craugastor laevissimus* | 3 | Hypothesized |
| *Eleutherodactylus locustus* | 3 | Hypothesized |
| *Craugastor merendonensis* | 3 | Hypothesized |
| *Eleutherodactylus olanchano* | 3 | Hypothesized |
| *Craugastor ranoides* | 4 | Hypothesized |
| *Eleutherodactylus richmondi* | 3 | Hypothesized |
| *Eleutherodactylus schmidti* | 3 | Hypothesized |
| *Gastrotheca pseustes* | 3 | Evidence |
| *Gastrotheca riobambae* | 3 | Hypothesized |
| *Isthmohlya tica* | 3 | Hypothesized |
| *Leiopelma archeyi* | 3 | Evidence |
| *Litoria nyakalensis* | 3 | Hypothesized |
| *Litoria raniformis* | 3 | Evidence |
| *Mixophyes iteratus* | 3 | N/A |
| *Nectophrynoides asperginis* | 3 | Evidence |
| *Quasipaa boulengeri* | 3 | N/A |
| *Quasipaa yunnanensis* | 3 | N/A |
| *Plectrohyla guatemalensis* | 4 | Hypothesized |
| *Taudactylus acutirostris* | 3 | Evidence |
| *Telmatobius niger* | 3 | Evidence |
