# Supplementary material for: Examining the Evidence for Chytridiomycosis in Threatened Amphibian Species
Source: PLoS One. 2011 Aug 3;6(8):e23150. doi: 10.1371/journal.pone.0023150 (PMC3149636; doi:10.1371/journal.pone.0023150)
Supplement: Table S3 — Critically endangered species used in advanced literature surveys to investigate lags between Red List assessments and the primarily scientific literature. Discrepancies existed for only 10 of 123 species. In all cases the Red List hypothesized the threat of chytridiomycosis whereas primary sources documented the presence of Bd in wild populations (although only five manifested in clinical disease). (DOC) [file pone.0023150.s003.doc]

Table S3

| **GENUS** | **SPECIES** | **# OF RETURNS** | **NEW EVIDENCE** | **SOURCE** |
| --- | --- | --- | --- | --- |
| *Agalychnis* | *moreletii* | 12 | YES | Felger et al. [1] |
| *Aromobates* | *leopardalis* | 8 | NO | - |
| *Atelopus* | *andinus* | 8 | NO | - |
| *Atelopus* | *angelito* | 2 | NO | - |
| *Atelopus* | *arsyecue* | 4 | NO | - |
| *Atelopus* | *arthuri* | 5 | NO | - |
| *Atelopus* | *balios* | 4 | NO | - |
| *Atelopus* | *boulengeri* | 12 | NO | - |
| *Atelopus* | *carauta* | 5 | NO | - |
| *Atelopus* | *carrikeri* | 8 | NO | - |
| *Atelopus* | *chocoensis* | 3 | NO | - |
| *Atelopus* | *chrysocorallus* | 5 | NO | - |
| *Atelopus* | *coynei* | 3 | NO | - |
| *Atelopus* | *ebenoides* | 5 | NO | - |
| *Atelopus* | *elegans* | 14 | NO | - |
| *Atelopus* | *erythropus* | 7 | NO | - |
| *Atelopus* | *eusebianus* | 5 | NO | - |
| *Atelopus* | *exiguus* | 10 | NO | - |
| *Atelopus* | *farci* | 3 | NO | - |
| *Atelopus* | *galactogaster* | 3 | NO | - |
| *Atelopus* | *glyphus* | 4 | NO | - |
| *Atelopus* | *guanujo* | 8 | NO | - |
| *Atelopus* | *guitarraensis* | 2 | NO | - |
| *Atelopus* | *laetissimus* | 8 | NO | - |
| *Atelopus* | *lozanoi* | 5 | NO | - |
| *Atelopus* | *lynchi* | 50 | NO | - |
| *Atelopus* | *mandingues* | 2 | NO | - |
| *Atelopus* | *mindoensis* | 3 | NO | - |
| *Atelopus* | *minutulus* | 4 | NO | - |
| *Atelopus* | *monohernandezii* | 2 | NO | - |
| *Atelopus* | *muisca* | 4 | NO | - |
| *Atelopus* | *nahumae* | 7 | NO | - |
| *Atelopus* | *nanay* | 15 | NO | - |
| *Atelopus* | *nepiozomus* | 2 | NO | - |
| *Atelopus* | *nicefori* | 10 | NO | - |
| *Atelopus* | *oxyrhynchus* | 13 | NO | - |
| *Atelopus* | *pedimarmoratus* | 6 | NO | - |
| *Atelopus* | *petriruizi* | 2 | NO | - |
| *Atelopus* | *pictiventris* | 4 | NO | - |
| *Atelopus* | *pinangoi* | 7 | NO | - |
| *Atelopus* | *planispina* | 3 | NO | - |
| *Atelopus* | *quimbaya* | 5 | YES | Lips et al. [2] |
| *Atelopus* | *reticulatus* | 9 | NO | - |
| *Atelopus* | *seminiferus* | 6 | NO | - |
| *Atelopus* | *sernai* | 4 | NO | - |
| *Atelopus* | *simulatus* | 3 | NO | - |
| *Atelopus* | *sonsonensis* | 3 | NO | - |
| *Atelopus* | *subornatus* | 9 | NO | - |
| *Atelopus* | *tamaensis* | 1 | NO | - |
| *Atelopus* | *walkeri* | 5 | NO | - |
| *Bromeliohyla* | *dendroscarta* | 2 | NO | - |
| *Centrolene* | *ballux* | 1 | NO | - |
| *Centrolene* | *gemmatum* | 0 | NO | - |
| *Centrolene* | *heloderma* | 2 | NO | - |
| *Charadrahyla* | *altipotens* | 3 | NO | - |
| *Charadrahyla* | *trux* | 2 | NO | - |
| *Craugastor* | *anciano* | 1 | NO | - |
| *Craugastor* | *cruzi* | 1 | NO | - |
| *Craugastor* | *emleni* | 1 | NO | - |
| *Craugastor* | *epochthidius* | 1 | NO | - |
| *Craugastor* | *fecundus* | 1 | NO | - |
| *Craugastor* | *greggi* | 4 | NO | - |
| *Craugastor* | *guerreroensis* | 2 | NO | - |
| *Craugastor* | *lineatus* | 6 | NO | - |
| *Craugastor* | *merendonensis* | 0 | NO | - |
| *Craugastor* | *olanchano* | 1 | NO | - |
| *Craugastor* | *omoaensis* | 1 | NO | - |
| *Craugastor* | *polymniae* | 4 | NO | - |
| *Craugastor* | *saltuarius* | 1 | NO | - |
| *Craugastor* | *stadelmani* | 2 | NO | - |
| *Craugastor* | *trachydermus* | 1 | NO | - |
| *Duellmanohyla* | *salvavida* | 1 | NO | - |
| *Ecnomiohyla* | *echinata* | 5 | NO | - |
| *Eleutherodactylus* | *orcutti* | 2 | NO | - |
| *Eleutherodactylus* | *schmidti* | 6 | NO | - |
| *Eleutherodactylus* | *semipalmatus* | 5 | NO | - |
| *Eleutherodactylus* | *symingtoni* | 2 | NO | - |
| *Eleutherodactylus* | *turquinensis* | 3 | NO | - |
| *Exerodonta* | *perkinsi* | 1 | NO | - |
| *Hyla* | *bocourti* | 7 | NO | - |
| *Hyloscirtus* | *ptychodactylus* | 1 | NO | - |
| *Hyloxalus* | *anthracinus* | 1 | NO | - |
| *Hyloxalus* | *delatorreae* | 2 | YES | Yanez-Munoz et al. [3] |
| *Hyloxalus* | *vertebralis* | 1 | NO | - |
| *Hypsiboas* | *cymbalum* | 0 | NO | - |
| *Leiopelma* | *archeyi* | 73 | YES | Smale [4] |
| *Leptophryne* | *cruentata* | 5 | YES | Kusrini et al. [5] |
| *Lithobates* | *omiltemanus* | 3 | NO | - |
| *Litoria* | *booroolongensis* | 12 | YES | Voyles et al. [6] |
| *Litoria* | *castanea* | 16 | NO | - |
| *Litoria* | *lorica* | 14 | YES | Puschendorf [7] |
| *Litoria* | *nyakalensis* | 12 | NO | - |
| *Litoria* | *piperata* | 11 | NO | - |
| *Litoria* | *spenceri* | 52 | YES | Speare & Berger [8] |
| *Mannophryne* | *caquetio* | 3 | NO | - |
| *Mannophryne* | *lamarcai* | 3 | NO | - |
| *Mannophryne* | *neblina* | 2 | NO | - |
| *Mannophryne* | *olmonae* | 11 | YES | Alemu et al. [9] |
| *Megastomatohyla* | *pellita* | 2 | NO | - |
| *Petropedetes* | *dutoiti* | 0 | NO | - |
| *Philoria* | *frosti* | 13 | NO | - |
| *Plectrohyla* | *calvicollina* | 4 | NO | - |
| *Plectrohyla* | *celata* | 2 | NO | - |
| *Plectrohyla* | *chrysopleura* | 2 | NO | - |
| *Plectrohyla* | *hartwegi* | 8 | NO | - |
| *Plectrohyla* | *hazelae* | 4 | NO | - |
| *Plectrohyla* | *pachyderma* | 3 | NO | - |
| *Plectrohyla* | *pycnochila* | 3 | NO | - |
| *Plectrohyla* | *tecunumani* | 1 | NO | - |
| *Plectrohyla* | *teuchestes* | 1 | NO | - |
| *Plectrohyla* | *thorectes* | 3 | NO | - |
| *Pristimantis* | *albericoi* | 0 | NO | - |
| *Pristimantis* | *lichenoides* | 9 | NO | - |
| *Pseudophryne* | *corroboree* | 28 | YES | Hunter et al. [10] |
| *Ptychohyla* | *dendrophasma* | 2 | NO | - |
| *Ptychohyla* | *macrotympanum* | 3 | NO | - |
| *Ranitomeya* | *abdita* | 2 | NO | - |
| *Rhinoderma* | *rufum* | 6 | NO | - |
| *Taudactylus* | *rheophilus* | 10 | NO | - |
| *Telmatobius* | *culeus* | 12 | NO | - |
| *Telmatobius* | *gigas* | 7 | NO | - |
| *Telmatobius* | *pefauri* | 0 | NO | - |
| *Telmatobius* | *zapahuirensis* | 0 | NO | - |

**SUPPORTING INFORMATION REFERENCES**

1. Felger J, Enssle J, Mendez D, Speare R (2007) Chytridiomycosis in El Salvador. Salamandra 43: 122-127.

2. Lips KR, Diffendorfer J, Mendelson III JR, Sears MW (2008) Riding the wave: Reconciling the roles of disease and climate change in amphibian declines. PLoS Biol 6: doi:101371/journalpbio0060072.

3. Yanez-Munoz MH, Meza-Ramos P, Altamirano B M, Castro M C (2010) Estado poblacional de una de rana nodriza (Anura: Dendrobatidae: *Hyloxalus delatorreae*), criticamente amenazada, en los Andes Norte de Ecuador. Boletin Tecnico 9 Serie Zoologica: 38-64.

4. Smale A (2006) Archey's frog emergency translocation - DOCDM-28159. Unpublished report. Department of Conservation, Waikato Conservancy, Hamilton.

5. Kusrini MD, Skerratt LF, Garland S, Berger L, Endarwin W (2008) Chytridiomycosis in frogs of Mount Gede Pangrango, Indonesia. Dis Aquat Org 82: 187-194.

6. Voyles J, Richards-Hrdlicka K, Cashins SD, Rosenblum EB, Hyatt AD, Berger L, Skerratt LF (2010) *Batrachochytrium dendrobatidis*: requirement for further isolate collection and archiving. Dis Aquat Org 92:109-112.

7. Puschendorf R (2009) Environmental effects on a host-pathogen system: Frogs and *Batrachochytrium dendrobatidis* in wet and dry habitats. Ph.D. thesis, James Cook University.

8. Speare R, Berger L (2005) Chytridiomycosis in amphibians in Australia. http://www.jcu.edu/au/school/phtm/PHTM/frogs/chyspec.htm.

9. Alemu JB, Cazabon MNE, Dempewolf L, Hailey A, Lehtinen RM, et al. (2008) Presence of chytrid fungus Batrachochytrium dendrobatidis in populations of critically endangered frog *Mannophyrne olmonae* in Tobago West Indies. EcoHealth 5: 34-39.

10. Hunter DA, Speare R, Marantelli G, Mendez D, Pietsch R, Osborne W (2009) Presence of the amphibian chytrid fungus *Batrachochytrium dendrobatidis* in threatened corroboree frog populations in the Australian Alps. Dis Aquat Org 92: 209-216.
